# Association between *Clostridioides difficile* Test Positivity and Colorectal Cancer Incidence in a Multisite Hospital-Based Retrospective Cohort Analysis

**DOI:** 10.1158/2767-9764.CRC-25-0606

**Published:** 2026-04-14

**Authors:** Samara Rifkin, Sean M. Anderson, Xingyu Chen, Kelly Gebo, Eili Klein, Nicholas O. Markham, Matthew Robinson, Krishna Rao, Cynthia L. Sears

**Affiliations:** 1 Division of Gastroenterology/Hepatology, University of Michigan Medicine, Ann Arbor, Michigan.; 2 Division of Gastroenterology, John D. Dingell VA Medical Center, Wayne State University School of Medicine, Detroit, Michigan.; 3 Division of Infectious Diseases, Johns Hopkins Medicine, Baltimore, Maryland.; 4 Johns Hopkins University School of Medicine, Baltimore, Maryland.; 5 Department of Emergency Medicine, Johns Hopkins University School of Medicine, Baltimore, Maryland.; 6 Department of Medicine, Vanderbilt University Medical Center, Nashville, Tennessee.; 7 Division of Infectious Diseases, University of Michigan Medicine, Ann Arbor, Michigan.

## Abstract

Sporadic colorectal cancer remains a significant driver of worldwide morbidity and mortality. Environmental factors associated with colorectal cancer are increasingly well-described and now include generalized colonic dysbiosis and individual enteric bacteria. *Clostridioides difficile* is one such species, with recent mouse model work suggesting prolonged exposure to *C. difficile* toxin B is conducive to colonic tumorigenesis. However, there is a dearth of real-world human evidence linking *C. difficile* exposure and colorectal cancer. Herein, we analyzed a multicenter, longitudinal, electronic health record–based dataset to test the association between *C. difficile* test positivity and the risk of incident colorectal cancer utilizing unadjusted and multivariable (controlled for clinical conditions independently associated with colorectal cancer development) Cox proportional hazard modeling to compare *C. difficile* exposed and nonexposed cohorts. We found that individuals who tested recurrently positive for *C. difficile* had a significantly increased risk of incident colorectal cancer [adjusted HR (aHR) 2.05 (95% confidence interval, 1.27–3.29)] compared with those who tested positive only once [aHR 0.70 (0.45–1.10)] or never. Furthermore, we found potential trends that the effect of *C. difficile* test positivity on the risk of incident colorectal cancer was stronger amongst females compared with males. These findings help translate emerging mouse model work on *C. difficile*–influenced colorectal tumorigenesis and lay groundwork for more substantial human investigations into this connection. These findings also may begin to help guide the personalized deployment of novel fecal microbiota–based therapies designed to interrupt the life cycle of *C. difficile* within the gut of human hosts and, potentially, prevent long-term health sequelae of chronic *C. difficile* infection.

**Significance:**

These findings help translate emerging mouse model work on *C. difficile*–influenced colorectal tumorigenesis and lay groundwork for more substantial human investigations into this connection. These findings also may begin to help guide the personalized deployment of novel fecal microbiota–based therapies designed to interrupt the life cycle of *C. difficile* within the gut of human hosts and, potentially, prevent long-term health sequelae of chronic *C. difficile* infection.

## Introduction

Sporadic colorectal cancer remains the second leading cause of cancer morbidity and mortality worldwide (1). Risk factors for sporadic colorectal cancer include both nonmodifiable (e.g., older age, male sex, and Black race) and modifiable (i.e., environmental) variables (2); the latter represents a critical area for study given the potential for disease prevention. Environmental factors contributing to colorectal cancer are complex, but there is emerging evidence that both generalized gut dysbiosis as well as individual enteric bacteria may contribute to colonic tumor formation (3). Dysbiosis and the overgrowth of certain potentially tumorigenic enteric bacteria are mediated by many factors, including diet, tobacco/alcohol use, and obesity, all of which have been independently shown to be linked to colorectal cancer development (2). Importantly, oral antibiotic use, an established precipitant of gut dysbiosis, has been associated with an increased risk of colorectal cancer (4–6).

Oral antibiotics also strongly predispose patients to infection with *Clostridioides difficile*, a diarrheal pathogen transmitted via the fecal–oral route with spores that are commonly encountered in the environment, both in the community and healthcare settings. Asymptomatic carriage of *C. difficile* occurs in ∼10% of the “healthy” population (7) and is most frequent in infants and hospitalized patients (8). *C. difficile* infection (CDI) is defined as a clinically significant diarrheal illness that occurs upon germination of colonizing *C. difficile* spores and production of bacterial toxins. CDI occurs in more than 500,000 people per year in the United States alone (9), and the clinical syndrome can vary from a mild diarrheal illness to fulminant and life-threatening colitis and sepsis. In recent years, female sex has been implicated as a risk factor for CDI (10), though the mechanism and long-term health consequences of this association remain unclear.

Although CDI remains a significant public health concern given a rise in community-onset and recurrent cases in recent decades (11), little is known about the natural history of *C. difficile* colonization and persistence following initial diagnosis and treatment attempts. However, there is evidence that some patients can continue to shed spores for over a month after initial treatment of CDI, potentially leading to continued long-term exposure to spores after symptoms have abated (12). Unexpectedly, recent mouse model data showed that chronic colonization with toxigenic strains of *C. difficile* promotes colonic tumorigenesis (13). A notable nuance in this conclusion was the need for prolonged exposure to intraluminal *C. difficile* toxin B (TcdB), the main virulence factor of *C. difficile* (14), for the procarcinogenic effect. The linkage between CDI and human colorectal cancer has not yet been thoroughly investigated. Taxonomic analyses of tissue in sporadic colorectal cancer have identified *C. difficile* enrichment (15), and several recent epidemiologic publications with limited study designs found conflicting results (16, 17). Specifically, these single-database studies relying on claims-based data found both increased and decreased colorectal cancer risk following an International Classification of Disease (ICD)-10 diagnosis of CDI, but these studies were likely limited by exposure misclassification and lack of temporal separation. In our study, we combined longitudinal data from two large institutions to test the association between a positive stool toxin assay for *C. difficile* and incident development of colorectal cancer among adults.

## Materials and Methods

### Study design, data extraction, and cohort construction

We performed a retrospective cohort study utilizing longitudinal medical data from two large academic medical institutions. University of Michigan Medicine (MM) patient-level health data were obtained from a data repository containing demographics, anthropometric data, ICD-9 and ICD-10 diagnosis coding, laboratory testing values, and medication prescriptions. Johns Hopkins Medicine (JHM) patient-level health data were obtained from a repository collected under the Infectious Diseases Precision Medicine Center of Excellence, a dataset which contains ICD-10 diagnosis coding, microbiological test results, medication order and administration records, abstracted social and demographic history, and surgical and medical histories collected from the electronic health record (EHR) of every patient within JHM who ever received a positive microbiologic test result. Local Institutional Review Board approval was obtained from each site to access data, which were fully deidentified prior to sharing between sites.

Each dataset was queried for all individuals 18 years of age and older who had completed stool-based testing for *C. difficile* within the timeframe captured by the respective database. For MM, the timeframe was between January 1, 2000, and August 2, 2023. For JHM, the timeframe was between January 1, 2016, and August 15, 2024. We used the date of the first stool-based C. *difficile* testing to define the date of cohort entry (index date). For both sites, individuals were excluded if they were documented as deceased within 365 days after entry or if they did not have a follow-up encounter of any type within the system at least 365 days after their first *C. difficile* test. For JHM, ICD-10 codes were queried for the presence of any code compatible with a diagnosis of colorectal cancer (C18.0-7, C19, and C20). For MM, prior colorectal cancer diagnoses were identified through the University of Michigan Rogel Cancer Center Registry. Individuals were excluded if their earliest date of any colorectal cancer diagnosis code was <365 days after entry of *C. difficile* test (including all available information prior to cohort entry). We also excluded individuals with a history of total colectomy (Current Procedural Terminology codes 44150, 44151, 44156–44158, and 44210–4421) prior to baseline.

The individuals remaining in the dataset after application of these exclusion criteria were classified as *C. difficile*–exposed if their first *C. difficile* test contained a positive result (including isolated *tcdb* nucleic acid amplification test positivity as part of a two-step testing algorithm) and nonexposed if not. For individuals who initially tested negative and subsequently had a positive test for *C. difficile* prior to database exit, they were crossed over from unexposed to exposed groups; their follow-up time before the positive test was counted as unexposed time, and their follow-up time after the positive test was counted as exposed time.

Finally, individuals who contributed any exposed follow-up time were categorized using a binary definition (never or ever) and nominal definition based on the number of positive *C. difficile* tests they had; if they had no positive *C*. *difficile* test, they were categorized as CD = 0; if only one positive test, CD = 1; and if at least one subsequent *C. difficile* test had a positive result ≥30 days after a previous positive test, they were classified as CD >1. This was based on the histogram showing significant skew and zero inflation with most individuals having no exposure and most patients with *C. difficile* positivity having only one positive test, with very few having two or more positive tests. Similar to the negative to positive crossover as described above, in the nominal analysis, patients contribute time to multiple nominal exposure definitions with each additional infection (0, 1, or >1).

### Covariates

Potential confounding risk factors were selected based on known clinical understanding of *C. difficile* and colon cancer pathogenesis. These variables included age at *C. difficile* testing, sex, obesity, diabetes mellitus (DM), preceding antibiotic use, preceding proton pump inhibitor use, history of inflammatory bowel disease (IBD), and family history of colon cancer. Most of these metrics were assessed by querying the databases for ICD-10 codes compatible with these risk factors at the time of cohort entry. Age and sex were abstracted from demographic data for JHM and MM. At MM, body mass index (BMI) was abstracted from anthropometric data, and obesity was defined as BMI >30 kg/m^2^. At JHH, BMI data were not readily available, so ICD-10 codes for obesity were used instead. For both sites, preceding antibiotic use was determined by a query of medication records for all oral or intravenous antibiotics except for oral vancomycin or fidaxomicin, as these are treatments for CDI and not classic exposures, within 90 days prior to cohort entry. Dietary and behavioral factors (such as alcohol or tobacco use) are known risk factors for colorectal cancer; however, given that these variables are not reliably coded into EHR data, they were not included in this study’s analysis.

### Colorectal cancer outcomes

The primary outcome was incident colorectal cancer >365 days after the index date, which was ascertained using the MM cancer registry or by acquisition of a new ICD-10 code compatible with colorectal cancer (JHM). Colorectal cancer diagnosis was also stratified into right (cecum, ascending colon, and hepatic flexures), left (transverse colon, splenic flexure, descending colon, and sigmoid), and rectal (rectum and rectosigmoid junction) locations based on ICD-10 code if site-specific information was available (94% available for MM, 78% available for JHM).

### Statistical analysis

Individuals were compared by CD exposure status (exposed vs. unexposed cohorts as above) using *t* test and *χ*^2^ tests for continuous and categorical variables. We performed survival analysis to examine the effect of exposed versus unexposed status and incident colorectal cancer outcomes. Participants contributed time as either exposed or unexposed depending on whether the index laboratory test was positive or negative. Those who tested positive at any later date after an initial negative test began to contribute to exposed time on the date of the positive test. Participants were followed from the date of their entry *C. difficile* test until they were diagnosed with colorectal cancer, death, or censoring (last encounter date in the system), whichever occurred first.

We used unadjusted and multivariable multilevel random effects Cox proportional analysis to estimate the hazard ratio (HR) of incident colorectal cancer, right colon, left colon, and rectal cancer associated with exposed status (both binary exposure definition and nominal definition of exposed status) and 95% confidence intervals (CI) of colorectal cancer. The model incorporated random intercepts for site-level (MM and JHM) effects, allowing the relationship between exposed status and colorectal cancer risk to vary across sites. Predictors of incident colorectal cancer, estimates, and 95% CIs were derived using a Cox model. The potential confounders included in the final model were selected based on prior epidemiologic literature on risk factors for colorectal cancer (2) and availability of variables at both clinical sites. The final multivariable Cox model included age, sex, race, DM, family history of colorectal cancer (FHCRC), and personal history of IBD. We decided not to include antibiotic use into the model because a minority of individuals with CDI had prior recorded antibiotic use, and we assumed the variable must be missing information. Models were also stratified by sex. Interactions between CD status and sex were investigated by creating a cross product and applying a likelihood ratio test. Analyses were also conducted using 1-, 3-, and 5-year time-lag intervals between exposure and outcome, excluding colorectal cancer events occurring prior to each respective lag period using the MM cohort only. Proportional hazards assumptions were tested by including an interaction term between CD exposure and follow-up time in a Cox regression model. All analyses were conducted using R (version 4.4.2), with the survival package (version 3.8.3).

## Results

### Cohort characteristics and colorectal cancer incidence

Overall, 100,181 individuals contributed 1,045,083 total person-years of follow-up (Table 1). Seventy-nine percent of the individuals and 91% of the follow-up time were contributed by the MM cohort. At baseline, the median age of the MM cohort was 55 years, 9.5% were Black, and 54% were female, whereas the JHM cohort was older with median age 62 years, 29% were Black, and 57% were female. Patients from MM were more likely to have IBD, DM, and a FHCRC compared with JHM. Of all the patients tested for *C. difficile*, 15% and 24% tested positive in the MM and JHM cohorts, respectively (17% overall positivity rate). Patients exposed to C. *difficile* were more likely to be female, older, carry a diagnosis of IBD or DM, and have a recent history of antibiotic use. During the follow-up time, there were 254 incident colorectal cancer cases: 165 (65%) from MM and 89 (35%) from JHM; 41 (16%) of these incident colorectal cancer cases were recorded in the *C. difficile*–exposed cohort. A breakdown of colorectal cancer by anatomic location is provided in Table 1. The overall incidence rate of colorectal cancer per 1,000 patient-years was 0.18 and 0.26 in the exposed and nonexposed cohorts, respectively.

**Table 1.** Baseline demographics of individuals according to CD exposure, MM (2000–2021), JHM (2016–2024).

Characteristic	Total			MM			JHM		
	No Hx of CD	Hx of CD	*P* value^a^	No Hx of CD	Hx of CD	*P* value^a^	No Hx of CD	Hx of CD	*P* value^a^
​	*N* = 83,397	*N* = 16,784	​	*N* = 67,323	*N* = 11,599	​	*N* = 16,074	*N* = 5,185	​
Age	56 (38, 69)^b^	57 (38, 70)^b^	<0.001	54 (35, 67)^b^	55 (34, 67)^b^	0.1	62 (48, 73)^b^	62 (48, 74)^b^	0.6
Sex	​	​	0.01	​	​	0.6	​	​	<0.001
Female	45,080 (54%)	9,248 (55%)	​	36,127 (54%)	6,252 (54%)	​	8,953 (56%)	2,996 (58%)	​
Male	38,313 (46%)	7,528 (45%)	​	31,192 (46%)	5,339 (46%)	​	7,121 (44%)	2,189 (42%)	​
DM	17,492 (21%)	3,885 (23%)	<0.001	14,500 (22%)	2,976 (26%)	<0.001	2,992 (19%)	912 (18%)	0.1
IBD	13,240 (16%)	3,280 (20%)	<0.001	12,626 (19%)	2,976 (26%)	<0.001	614 (3.8%)	304 (5.9%)	<0.001
FHCRC	3,144 (3.8%)	640 (3.7%)	0.8	2,778 (4.1%)	521 (4.5%)	0.07	366 (2.3%)	119 (2.3%)	>0.9
Obesity	​	​	<0.001	​	​	<0.001	​	​	0.2
No	47,229 (56%)	11,624 (67%)	​	31,416 (47%)	6,221 (54%)	​	​	​	​
Yes	17,571 (21%)	3,334 (20%)	​	16,803 (25%)	3,110 (27%)	​	768 (4.8%)	224 (4.3%)	​
Unknown	19,171 (23%)	2,275 (13%)	​	19,171 (28%)	2,275 (20%)	​	​	​	​
Antibiotics	36,240 (43%)	8,965 (53%)	<0.001	25,879 (38%)	5,690 (49%)	<0.001	10,361 (64%)	3,275 (63%)	0.1
PPI	19,746 (24%)	4,096 (24%)	0.05	17,428 (26%)	3,577 (31%)	<0.001	2,318 (14%)	519 (10%)	<0.001
Race	​	​	<0.001	​	​	<0.001	​	​	0.01
Caucasian	65,638 (79%)	12,784 (76%)	​	55,853 (83%)	9,575 (83%)	​	9,978 (61%)	3,209 (62%)	​
Black	10,877 (13%)	2,709 (16%)	​	6,377 (9.5%)	1,223 (11%)	​	4,500 (28%)	1,488 (29%)	​
Other	6,787 (8.1%)	1,272 (7.6%)	​	5,093 (7.6%)	803 (6.9%)	​	1,694 (11%)	469 (9.1%)	​
CRC	213 (0.3%)	41 (0.3%)	>0.9	139 (0.2%)	26 (0.2%)	0.8	74 (0.5%)	15 (0.3%)	0.1
Right colon	64 (<0.1%)	15 (<0.1%)	0.6	53 (<0.1%)	8 (<0.1%)	0.9	11 (<0.1%)	7 (0.1%)	0.2
Left colon	59 (<0.1%)	11 (<0.1%)	0.8	45 (<0.1%)	10 (<0.1%)	0.6	14 (<0.1%)	1 (<0.1%)	0.1
Rectum	65 (<0.1%)	17 (0.1%)	0.3	34 (<0.1%)	8 (<0.1%)	0.6	31 (0.2%)	9 (0.2%)	0.9
PY	4 (1.2, 7.7)	4 (1.6, 7.2)	0.007	3.9 (0.8, 8.7)	4.1 (1, 8.9)	>0.9	4.07 (2.4, 5.8)	3.97 (2.3, 5.7)	0.005

Abbreviations: CRC, colorectal cancer; PPI, proton pump inhibitor; PY, person-years of follow-up.

Baseline demographics are broken down into three groups: MM, JHM, and combined (“total”) cohorts. Each group is then subdivided into exposed (Hx of CD) and nonexposed (no Hx of CD) subcohorts for comparison. *P* values are determined by Wilcoxon rank-sum test for continuous variables or Pearson *χ*^2^ test (for proportions).

a Welch two-sample *t* test; Pearson *χ*^2^ test.

b Median (Q1, Q3); *n* (%).

### Multivariate modeling for association of *C. difficile* exposure to colorectal cancer risk

In both the MM and JHM cohorts, *C. difficile* exposure was not significantly associated with higher risk of colorectal cancer [adjusted HR (aHR), 1.43 (95% CI, 0.94–2.18) and aHR, 0.62 (95% CI, 0.36–1.08), respectively; Table 2]. Similarly, when combined, *C. difficile* exposure was not significantly associated with higher risk of colorectal cancer [aHR, 1.01 (95% CI, 0.72–1.42)]. Stratified by sex, *C. difficile* exposure was not significantly associated with increased risk of colorectal cancer among women overall [aHR, 1.22 (95% CI, 0.79–1.87)], nor or in either the MM cohort [aHR, 1.67 (95% CI, 0.95–2.94)] nor the JHM cohort [aHR, 0.85 (95% CI, 0.45–1.60)]. For males, *C. difficile* exposure was significantly associated with reduced likelihood for colorectal cancer in the JHM cohort [aHR, 0.30 (95% CI, 0.09–0.98)] but was not significantly associated with increased risk in the MM male cohort [aHR, 1.20 (95% CI, 0.63–2.27)]. Combined, the cohorts demonstrated no significant association in males. The interaction term of the cross product between *C. difficile* exposure and sex was also not significant (*P* = 0.10).

**Table 2.** Association between any CD infection and colorectal cancer incidence by sex, MM (2000–2021), JHM (2016–2024).

​	Total			MM			JHM		
	Events/PY	HR (95% CI)^a^	*P* value^b^	Events/PY	HR (95% CI)^a^	*P* value^b^	Events/PY	HR (95% CI)^a^	*P* value^b^
All sex	​	​	​	​	​	​	​	​	​
**CD**	**41**/225,874	1.01 (0.72–1.42)	>0.9	**26**/204,439	1.43 (0.94–2.18)	0.10	**15**/21,435	0.62 (0.36–1.08)	0.09
**Never**	**213**/819,209	Ref	—	**139**/751,312	Ref	—	**74**/67,898	Ref	—
Male	​	1.21 (0.94–1.55)	0.14	​	1.39 (1.02–1.89)	0.04	​	0.91 (0.59–1.41)	0.7
Age	​	1.04 (1.03–1.05)	<0.001	​	1.05 (1.04–1.06)	<0.001	​	1.01 (1–1.02)	0.10
DM	​	1.52 (1.17–1.98)	0.002	​	1.46 (1.07–2)	0	​	1.52 (0.93–2.47)	0.09
IBD	​	1.41 (1.02–1.94)	0.036	​	1.25 (0.88–1.7)	0.18	​	3.11 (1.61–6.02)	<0.001
FHCRC	​	2.84 (1.99–4.06)	<0.001	​	2.48 (1.33–4)	0.003	​	4.11 (1.97–8.56)	<0.001
Black	​	1.17 (0.82–1.66)	0.4	​	1.51 (0.93–2.45)	0.11	​	0.83 (0.53–1.32)	0.40
Other	​	0.69 (0.38–1.24)	0.2	​	0.80 (0.37–1.70)	0.59	​	0.54 (0.22–1.33)	0.17
Female									
**CD**	**27**/135,221	1.22 (0.79–1.87)	0.41	**15**/122,736	1.67 (0.95–2.94)	**0.07**	**12**/12,485	0.85 (0.45–1.60)	0.60
**Never**	**110**/470,480	Ref	—	**68**/432,055	Ref	—	**42**/38,426	Ref	—
Age	​	1.04 (1.03–1.05)	<0.001	​	1.06 (1.04–1.07)	<0.001	​	1.01 (0.99–1.02)	0.30
DM	​	1.73 (1.21–2.48)	0.003	​	1.44 (0.92–2.26)	0.11	​	2.33 (1.30–4.19)	0.005
IBD	​	1.35 (0.87–2.09)	0.2	​	1.04 (0.63–1.70)	0.9	​	4.15 (1.94–8.86)	<0.001
FHCRC	​	2.81 (1.75–4.52)	<0.001	​	2.42 (1.38–4.24)	0.002	​	3.77 (1.62–8.79)	0.002
Black	​	1.21 (0.77–1.90)	0.4	​	1.46 (0.74–2.85)	0.3	​	0.89 (0.50–1.62)	0.73
Other	​	0.66 (0.29–1.52)	0.3	​	0.93 (0.34–2.56)	0.9	​	0.39 (0.10–1.62)	0.20
Male									
**CD**	**14**/90,653	0.76 (0.43–1.34)	0.3	**11**/81,703	**1.20 (0.63–2.27)**	**0.61**	**3**/8,950	0.30 (0.09–0.98)	0.05
**Never**	**103**/348,729	Ref	—	**71**/319,257	Ref	—	**32**/29,472	Ref	—
Age	​	1.04 (1.03–1.05)	<0.001	​	1.05 (1.03–1.06)	<0.001	​	1.01 (0.99–1.04)	0.15
DM	​	1.31 (0.89–1.93)	0.2	​	1.50 (0.96–2.34)	0.07	​	0.68 (0.27–1.71)	0.39
IBD	​	1.49 (0.93–2.38)	0.10	​	1.51 (0.92–2.47)	0.11	​	1.58 (0.39–6.38)	0.70
FHCRC	​	2.88 (1.67–4.95)	<0.001	​	2.58 (1.44–4.62)	0.001	​	4.97 (1.17–21.10)	0.03
Black	​	1.09 (0.63–1.90)	0.8	​	1.56 (0.77–3.15)	0.22	​	0.67 (0.29–1.54)	0.33
Other	​	0.72 (0.31–1.65)	0.4	​	0.67 (0.21–2.12)	0.51	​	0.69 (0.21–2.27)	0.51

The association between *C. difficile* exposure (CD) and incident (>1 year after index date) development of colorectal cancer (CRC) is tested. “Events” indicate discrete incident diagnoses of CRC (number of positive events for each subgroup has been bolded), and “PY” indicates total patient-years of follow-up in each subcohort. Again, MM and JHM are analyzed individually and combined (“total”). Furthermore, each dataset is stratified by sex. “Never” indicates the nonexposed group (i.e., never tested positive for *C. difficile*). HRs and 95% CIs shown for the “CD” rows are calculated via multivariable Cox proportional hazard modeling adjusting for age, sex, DM, obesity, IBD, FHCRC, and preceding antibiotic use. The nonbolded variables are also tested for univariate associations with incident CRC development.

a HRs and 95% CIs are modeled with Cox proportional hazard.

b Multivariate Cox proportional hazard adjusted for age, sex (except for the restricted analyses), DM, IBD, FHCRC, and race.

### *C. difficile* nominal dose effect and colorectal cancer risk

We then assessed the association between multiple positive C. *difficile* tests and the risk of colorectal cancer (Table 3). Of the 16,784 individuals in the exposed cohort, 3,815 (22%) were classified as multiply exposed (i.e., CDI >1); 19 of the 41 colorectal cancer cases (46%) in the overall exposed cohort were identified in the CD >1 subgroup. Having two or more positive *C. difficile* assays spaced at least 30 days apart was significantly associated with an increased risk of colorectal cancer compared with nonexposed individuals [aHR, 2.05 (95% CI, 1.27–3.29)], whereas a history of only one positive *C. difficile* assay did not show an increased risk [aHR, 0.70 (95% CI, 0.45–1.10)]. In the MM cohort, this positive association was stronger [aHR, 2.97 (95% CI, 1.64–5.40)], whereas in the JHM cohort, the association was weaker and not statistically significant [aHR, 1.23 (95% CI, 0.56–2.67)]. When adjusting for the magnitude of *C. difficile* exposure, we found a significant association between exposure and colorectal cancer in females [aHR for females, 2.43 (95% CI, 1.36–4.37)] but not in males [aHR for males, 1.52 (95% CI, 0.66–3.49)]. These associations were again stronger in the MM cohort.

**Table 3.** Association between nominal dose effect of CD exposure (0, 1, and >1) and colorectal cancer incidence by sex, MM (2000–2021), JHM (2016–2024).

​	Total			MM			JHM		
	Events/PY	HR (95% CI)^a^	*P* value^b^	Events/PY	HR (95% CI)^a^	*P* value^b^	Events/PY	HR (95% CI)^a^	*P* value^b^
All sex	​	​	​	​	​	​	​	​	​
**CD = 0**	**213**/819,209	Ref	—	**139**/751,311	Ref	—	**74**/67,898	Ref	—
**CD = 1**	**22**/140,775	0.70 (0.45–1.10)	0.12	**14**/124,082	0.99 (0.57–1.72)	>0.9	**8**/16,693	0.44 (0.21–0.90)	0.03
**CD > 1**	**19**/85,097	2.05 (1.27–3.29)	0.003	**12**/80,357	2.97 (1.64–5.40)	<0.001	**7**/4,740	1.23 (0.56–2.67)	0.6
Male	​	1.21 (0.95–1.55)	0.13	​	1.39 (1.02–1.90)	0.035	​	0.92 (0.60–1.41)	0.7
Age	​	1.04 (1.03–1.05)	<0.001	​	1.05 (1.04–1.06)	<0.001	​	1.01 (1–1.02)	0.13
DM	​	1.50 (1.15–1.96)	0.002	​	1.45 (1.06–1.99)	0.021	​	1.50 (0.91–2.45)	0.11
IBD	​	1.38 (1–1.91)	0.047	​	1.22 (0.86–1.73)	0.3	​	3.10 (1.58–6.09)	0.001
FHCRC	​	2.83 (1.98–4.04)	<0.001	​	2.47 (1.65–3.70)	<0.001	​	4.04 (1.94–8.41)	<0.001
Black	​	1.18 (0.83–1.67)	0.4	​	1.51 (0.93–2.46)	0.10	​	0.83 (0.51–1.36)	0.5
Other	​	0.70 (0.39–1.25)	0.2	​	0.80 (0.37–1.71)	0.6	​	0.54 (0.22–1.35)	0.2
Female									
**CD = 0**	**110**/470,480	Ref	—	**68**/432,054	Ref	—	**42**/38,426	Ref	—
**CD = 1**	**14**/81,658	0.83 (0.48–1.47)	0.5	**7**/72,076	1.02 (0.47–2.22)	>0.9	**7**/9,582	0.67 (0.30–1.50)	0.3
**CD > 1**	**13**/53,563	2.43 (1.36–4.37)	0.003	**8**/50,660	3.90 (1.85–8.22)	<0.001	**5**/2,902	1.35 (0.53–3.42)	0.5
Age	​	1.04 (1.03–1.05)	<0.001	​	1.06 (1.04–1.07)	<0.001	​	1.01 (0.99–1.02)	0.4
DM	​	1.70 (1.19–2.44)	0.004	​	1.43 (0.91–2.25)	0.12	​	2.29 (1.27–4.15)	0.006
IBD	​	1.32 (0.85–2.04)	0.2	​	1 (0.61–1.64)	>0.9	​	4.12 (1.90–8.94)	<0.001
FHCRC	​	2.79 (1.74–4.48)	<0.001	​	2.41 (1.38–4.23)	0.002	​	3.69 (1.57–8.66)	0.003
Black	​	1.22 (0.78–1.93)	0.4	​	1.47 (0.75–2.87)	0.3	​	0.89 (0.49–1.64)	0.7
Other	​	0.67 (0.29–1.53)	0.3	​	0.94 (0.34–2.59)	>0.9	​	0.40 (0.10–1.65)	0.2
Male									
**CD = 0**	**103**/348,729	Ref	—	**71**/319,257	​	​	**32**/29,472	Ref	—
**CD = 1**	**8**/59,117	0.55 (0.27–1.14)	0.11	**7**/52,006	0.97 (0.45–2.12)	>0.9	**1**/7,111	0.12 (0.02–0.89)	0.04
**CD > 1**	**6**/31,535	1.52 (0.66–3.49)	0.3	**4**/29,697	2.05 (0.74–5.66)	0.2	**2**/1,838	**0.92 (0.22–3.86)**	0.99
Age	​	1.04 (1.03–1.05)	<0.001	​	1.05 (1.03–1.06)	<0.001	​	1.01 (1–1.04)	0.05
DM	​	1.30 (0.88–1.92)	0.2	​	1.49 (0.96–2.33)	0.076	​	0.62 (0.25–1.57)	0.30
IBD	​	1.47 (0.92–2.36)	0.11	​	1.49 (0.91–2.44)	0.12	​	1.30 (0.32–5.20)	0.70
FHCRC	​	2.86 (1.67–4.93)	<0.001	​	2.57 (1.44–4.60)	0.001	​	4.47 (1.07–18.7)	0.04
Black	​	1.09 (0.63–1.91)	0.8	​	1.56 (0.77–3.15)	0.2	​	0.63 (0.27–1.46)	0.30
Other	​	0.72 (0.32–1.66)	0.4	​	0.67 (0.21–2.12)	0.5	​	0.65 (0.20–2.17)	0.50

Abbreviation: PY, person-years of follow-up.

*C. difficile* exposure is stratified nominally as described in “Materials and Methods” to create three distinct exposure cohorts; these cohorts are then subjected to the same analyses described in Table 2.

a HRs and 95% CIs are modeled with Cox proportional hazard.

b Multivariate Cox proportional hazard adjusted for age, sex (except for the restricted analyses), DM, IBD, FHCRC, and race.

### Association between any *C. difficile* exposure and nominal *C. difficile* dose effect and colorectal cancer incidence by anatomic site

When evaluated by anatomic location broken down by proximal colon (i.e., right-sided), distal colon (i.e., left-sided), and rectal cancer, there was no significant association between binary *C. difficile* exposure and colorectal cancer (Tables 4 and 5). However, when the magnitude of *C. difficile* exposure was incorporated into the model, having multiple positive assays was significantly associated with proximal colon cancer [aHR 3.28 (95% CI, 1.55–6.93)] and rectal cancer [aHR 2.39 (95% CI, 1.14–5.03)] but not distal colon cancer [aHR 2.26 (95% CI, 0.90–5.71)]. For proximal colon cancer, the association was primarily driven by a strong positive correlation in the JHM cohort, whereas rectal cancer was more strongly associated with the MM cohort.

**Table 4.** Association between any CD exposure and colorectal cancer incidence by site (right colon, left colon, and rectum), MM (2000–2021), JHM (2016–2022).

​	Total			MM			JHM		
	Events/PY	HR (95% CI)^a^	*P* value^b^	Events/PY	HR (95% CI)^aa^	*P* value^b^	Events/PY	HR (95% CI)^a^	*P* value^b^
Right colon	​	​	​	​	​	​	​	​	​
**CDI**	**15**/226,301	1.39 (0.79–2.47)	0.3	**8**/204,866	1.14 (0.54–2.40)	0.7	**7**/21,435	1.94 (0.75–5.02)	0.2
**Never**	**64**/819,343	Ref	—	**53**/751,455	Ref	—	**11**/67,898	Ref	—
Male	​	1.10 (0.70–1.72)	0.7	​	1.12 (0.67–1.87)	0.7	​	1.11 (0.42–2.93)	0.8
Age	​	1.04 (1.03–1.06)	<0.001	​	1.05 (1.03–1.07)	<0.001	​	1.02 (0.99–1.05)	0.14
DM	​	1.27 (0.79–2.04)	0.3	​	1.45 (0.86–2.44)	0.2	​	0.50 (0.11–2.23)	0.4
IBD	​	1.22 (0.71–2.12)	0.5	​	1.16 (0.65–2.07)	0.6	​	2.59 (0.57–11.9)	0.2
FHCRC	​	3.73 (2.13–6.53)	<0.001	​	2.88 (1.53–5.44)	0.001	​	13 (4.16–41)	<0.001
Black	​	1.45 (0.77–2.71)	0.2	​	1.73 (0.81–3.67)	0.2	​	0.94 (0.33–2.72)	>0.9
Other	​	0.42 (0.10–1.72)	0.2	​	0.63 (0.15–2.60)	0.5	​	—	—
Left colon									
**CDI**	**11**/226,301	1.12 (0.58–2.15)	**0.69**	**10**/204,866	1.69 (0.85–3.36)	0.14	**1**/21,435	0.23 (0.03–1.74)	0.2
**Never**	**59**/819,343	Ref	—	**45**/751,455	Ref	​	**14**/67,898	Ref	—
Male	​	1.07 (0.66–1.72)	0.81	​	1.19 (0.70–2.03)	0.53	​	0.71 (0.24–2.11)	0.5
Age	​	1.05 (1.03–1.07)	<0.001	​	1.06 (1.04–1.07)	<0.001	​	1.02 (0.99–1.06)	0.2
DM	​	2.13 (1.31–3.44)	0.002	​	2.05 (1.19–3.48)	0.009	​	2.24 (0.75–6.70)	0.15
IBD	​	1.12 (0.61–2.06)	0.69	​	1.10 (0.59–2.07)	0.78	​	2.17 (0.28–16.9)	0.5
FHCRC	​	1.79 (0.85–3.76)	0.13	​	1.38 (0.59–3.24)	0.47	​	6.64 (1.47–30)	0.014
Black	​	1.06 (0.51–1.24)	0.91	​	1.30 (0.15–2.60)	0.51	​	0.66 (0.18–2.49)	0.5
Other	​	0.88 (0.32–2.43)	0.79	​	0.63 (0.15–2.60)	0.49	​	1.42 (0.31–6.56)	0.7
Rectum									
**CDI**	**17**/22,630	1.20 (0.70–2.07)	0.5	**8**/204,866	1.80 (0.83–3.92)	0.14	**9**/21,435	0.83 (0.40–1.76)	0.6
**Never**	**65**/819,343	Ref	—	**34**/751,455	Ref	—	**31**/67,898	Ref	—
Male	​	1.23 (0.80–1.91)	0.3	​	2.28 (1.22–4.24)	0.009	​	0.63 (0.32–1.24)	0.2
Age	​	1.02 (1.01–1.04)	<0.001	​	1.05 (1.03–1.07)	<0.001	​	1 (0.98–1.02)	0.8
DM	​	0.89 (0.52–1.53)	0.7	​	0.71 (0.35–1.42)	0.3	​	1.12 (0.49–2.57)	0.8
IBD	​	2.52 (1.47–4.32)	<0.001	​	1.90 (1–3.61)	0.051	​	4.90 (2.18–11)	<0.001
FHCRC	​	4.59 (2.56–8.23)	<0.001	​	3.71 (1.81–7.60)	<0.001	​	5.50 (2.13–14.2)	<0.001
Black	​	0.92 (0.49–1.71)	0.8	​	1.48 (0.52–4.21)	0.5	​	0.65 (0.30–1.40)	0.3
Other	​	0.82 (0.33–2.05)	0.7	​	1.49 (0.46–4.86)	0.5	​	0.45 (0.11–1.91)	0.3

Abbreviation: PY, person-years of follow-up.

Incident colorectal cancer (CRC) diagnoses are stratified by location (right colon, left colon, and rectum) as determined by ICD-10 coding (see “Materials and Methods”). *C. difficile* exposure (“CDI”) vs. nonexposure (“never”) is then tested for association with incident CRC by location using the same analyses described in Table 2.

a HRs and 95% CIs modeled with Cox proportional hazard.

b Multivariate Cox proportional hazard adjusted for age, sex, DM, IBD, FHCRC, and race.

**Table 5.** Association between nominal dose effect of CD exposure (0, 1, and >1) and colorectal cancer incidence by anatomic site (right colon, left colon, and rectum), MM (2000–2021), JHM (2016–2024).

​	Total			MM			JHM		
	Events/PY	HR (95% CI)^a^	*P* value^b^	Events/PY	HR (95% CI)^a^	*P* value^b^	Events/PY	HR (95% CI)^a^	*P* value^b^
Right colon	​	​	​	​	​	​	​	​	​
**CD = 0**	**64**/819,343	Ref	—	**53**/751,445	Ref	—	**11**/67,898	Ref	—
**CD = 1**	**7**/140,915	0.84 (0.38–1.85)	0.7	**4**/124,220	0.73 (0.27–2.03)	0.6	**3**/16,695	1.09 (0.30–3.93)	0.9
**CD > 1**	**8**/85,386	3.28 (1.55–6.93)	0.002	**4**/80,645	2.55 (0.91–7.10)	0.074	**4**/4,740	4.65 (1.46–14.8)	0.009
Male	​	1.11 (0.71–1.73)	0.7	​	1.13 (0.68–1.88)	0.6	​	1.12 (0.43–2.95)	0.8
Age	​	1.04 (1.03–1.06)	<0.001	​	1.05 (1.03–1.07)	<0.001	​	1.02 (0.99–1.05)	0.14
DM	​	1.25 (0.78–2.01)	0.4	​	1.44 (0.86–2.42)	0.2	​	0.47 (0.10–2.08)	0.3
IBD	​	1.18 (0.68–2.06)	0.5	​	1.13 (0.63–2.02)	0.7	​	2.45 (0.52–11.5)	0.3
FHCRC	​	3.71 (2.12–6.51)	<0.001	​	2.88 (1.52–5.44)	0.001	​	12.5 (3.97–39.2)	<0.001
Black	​	1.46 (0.78–2.74)	0.2	​	1.73 (0.81–3.69)	0.2	​	0.94 (0.32–2.75)	>0.9
Other	​	0.42 (0.10–1.73)	0.2	​	0.63 (0.15–2.61)	0.5	​	—	—
Left colon									
**CD = 0**	**59**/819,343	Ref	—	**45**/751,445	Ref	—	**14**/67,898	Ref	—
**CD = 1**	**6**/140,915	0.79 (0.34–1.84)	0.6	**5**/124,220	1.08 (0.43–2.74)	0.9	**1**/16,695	0.30 (0.04–2.32)	0.3
**CD > 1**	**5**/85,386	2.26 (0.90–5.71)	0.083	**5**/80,645	3.89 (1.52–9.95)	0.004	**0**/4,740	—	—
Male	​	1.07 (0.67–1.73)	0.8	​	1.20 (0.70–2.04)	0.5	​	0.71 (0.24–2.10)	0.5
Age	​	1.05 (1.03–1.07)	<0.001	​	1.06 (1.04–1.07)	<0.001	​	1.02 (0.99–1.06)	0.2
DM	​	2.11 (1.30–3.41)	0.002	​	2.03 (1.18–3.47)	0.010	​	2.26 (0.76–6.76)	0.14
IBD	​	1.11 (0.60–2.04)	0.7	​	1.07 (0.57–2.01)	0.8	​	2.17 (0.28–17)	0.5
FHCRC	​	1.78 (0.84–3.75)	0.13	​	1.38 (0.59–3.23)	0.5	​	6.72 (1.49–30.3)	0.013
Black	​	1.06 (0.51–2.21)	0.9	​	1.30 (0.55–3.07)	0.5	​	0.66 (0.17–2.48)	0.5
Other	​	0.88 (0.32–2.45)	0.8	​	0.64 (0.15–2.62)	0.5	​	1.42 (0.31–6.53)	0.7
Rectum									
**CD = 0**	**67**/819,343	Ref	—	**34**/751,445	Ref	—	**31**/69,145	Ref	—
**CD = 1**	**9**/140,915	0.83 (0.41–1.69)	0.6	**5**/124,220	1.47 (0.57–3.77)	0.4	**4**/16,901	0.73 (0.27–2.03)	0.6
**CD > 1**	**8**/85,386	2.39 (1.14–5.03)	0.022	**3**/80,645	2.93 (0.89–9.69)	0.78	**5**/4,782	2.55 (0.91–7.10)	0.074
Male	​	1.24 (0.80–1.93)	0.3	​	2.29 (1.23–4.25)	0.009	​	1.13 (0.68–1.88)	0.6
Age	​	1.02 (1.01–1.04)	<0.001	​	1.05 (1.03–1.07)	<0.001	​	1.05 (1.03–1.07)	<0.001
DM	​	0.88 (0.52–1.50)	0.6	​	0.70 (0.35–1.41)	0.3	​	1.44 (0.86–2.42)	0.2
IBD	​	2.47 (1.44–4.25)	0.001	​	1.86 (0.98–3.55)	0.059	​	1.13 (0.63–2.02)	0.7
FHCRC	​	4.55 (2.54–8.14)	<0.001	​	3.70 (1.80–7.57)	<0.001	​	2.88 (1.52–5.44)	0.001
Black	​	0.92 (0.49–1.72)	0.8	​	1.48 (0.52–4.20)	0.5	​	1.73 (0.81–3.69)	0.2
Other	​	0.82 (0.33–2.06)	0.7	​	1.49 (0.46–4.85)	0.5	​	0.63 (0.15–2.61)	0.5

Abbreviation: PY, person-years of follow-up.

Nominal *C. difficile* exposure stratification as described in Table 3 is tested for association with incident colorectal cancer diagnoses by colon location as described in Table 4.

a HRs and 95% CIs modeled with Cox proportional hazard.

b Multivariate Cox proportional hazard adjusted for age, sex, DM, IBD, FHCRC, and race.

### Time-lagged associations between nominal *C. difficile* exposure dose effect and colorectal cancer incidence

When evaluating the effect of time lag on the MM cohort, the overall association between *C. difficile* exposure on colorectal cancer incidence remained unchanged (Table 6). An increased risk of colorectal cancer incidence among patients with more than one prior *C. difficile* test persisted at all lags times, although CIs were wide. The association between a single prior positive *C. difficile* test and colorectal cancer incidence remained null.

**Table 6.** Time-lagged associations between nominal *C. difficile* exposure dose effect and colorectal cancer incidence, MM (2000–2021).

Characteristic	1-year lag			3-year lag			5-year lag		
	Events/PY	HR (95% CI)^a^	*P* value^b^	Events/PY	HR (95% CI)^a^	*P* value^b^	Events/PY	HR (95% CI)^a^	*P* value^b^
CD = 0	139/751,312	—	—	96/70,876	—	—	59/646,257	—	—
CD = 1	14/124,082	0.98 (0.57–1.70)	>0.9	10/116,629	1.03 (0.53–1.97)	>0.9	8/05,489	1.39 (0.66–2.91)	0.4
CD > 1	12/80,358	2.58 (1.42–4.66)	0.002	9/77,852	2.70 (1.36–5.37)	0.005	7/72,765	3.62 (1.64–7.99)	0.001
Male	​	1.36 (1–1.86)	0.049	​	1.41 (0.98–2.05)	0.065	​	1.67 (1.05–2.65)	0.03
Age	​	—	​	​	—	​	​	1.05 (1.03–1.06)	<0.001
DM	​	1.49 (1.09–2.05)	0.014	​	1.54 (1.05–2.25)	0.026	​	1.09 (0.67–1.78)	0.7
IBD	​	1.12 (0.79–1.59)	0.5	​	0.92 (0.60–1.42)	0.7	​	0.79 (0.45–1.37)	0.4
FHCRC	​	2.47 (1.65–3.70)	<0.001	​	2.82 (1.78–4.48)	<0.001	​	3.11 (1.80–5.38)	<0.001
Black	​	1.44 (0.89–2.34)	0.14	​	1.37 (0.77–2.46)	0.3	​	1.03 (0.44–2.40)	>0.9
Other	​	0.78 (0.37–1.67)	0.5	​	0.62 (0.23–1.68)	0.3	​	0.70 (0.22–2.24)	0.6

Abbreviation: PY, person-years of follow-up.

For the MM cohort, the association of *C. difficile* exposure on incident colorectal cancer development is examined at 1-, 3-, and 5-year lags using the same analyses as described in Table 2.

a HRs and 95% CIs modeled with Cox proportional hazard.

b Multivariate Cox proportional hazard adjusted for age, sex, DM, IBD, FHCRC, and race.

## Discussion

In this multicenter, retrospective cohort study, using EHR data, we identified an intriguing association between persistent/recurrent *C. difficile* assay positivity and the future risk of colorectal cancer. These results help advance the translation of mouse model studies associating this ubiquitous pathogen with the pathophysiology of colorectal cancer. Most notably, when controlling for other variables established as independent predictors of colorectal cancer, we found that persistent or recurrent *C. difficile* exposure (defined as at least a second positive test at least 30 days following the first positive test) was significantly associated with the later development of colorectal cancer compared with those who only experienced a single positive test and those who never tested positive for *C. difficile* with at least 365 days of follow-up. This effect was consistent in both databases examined, although only statistically significant in the MM dataset. Additionally, we found trends suggesting a sex-based effect on *C. difficile–*associated colorectal cancer in that female sex seemed to strengthen the risk that *C. difficile* positivity had on eventual diagnosis of colorectal cancer.

These findings differ from other recent publications investigating similar questions. One study (11) utilizing data from the Florida Medicaid system found that a diagnosis of CDI was associated with an increased risk of colorectal cancer development in the subsequent 4 years. However, this study constructed their *C. difficile* cohort using ICD-based diagnosis claims for CDI as opposed to the use of *C. difficile* test results which we implemented in this present study. As a result, only 0.3% of the individuals analyzed were classified as exposed (compared with 17% in our study), which is likely a vast underestimation of true *C. difficile* exposure. Another more comprehensive study (12) used a national claims-based database to match CDI and non-CDI patients 1:1, based on ICD-10 coding and National Drug Codes for antibiotics used to treat CDI. This study, interestingly, found opposite results, that CDI exposure was protective against colorectal cancer (except for obese patients, in whom CDI exposure increased the risk of colorectal cancer compared with nonexposed obese patients). That study, however, did not apply any time-based cutoffs for colorectal cancer diagnosis following CDI exposure, meaning that colorectal cancer diagnoses could have been essentially concurrent with CDI episodes; this calls any potential associations of *C. difficile* with the natural history of colorectal cancer into question. Furthermore, the matching was performed on many variables, including age, sex, Charlson comorbidity index, CDI treatment, and obesity, that may have inadvertently been overlooked as modifiers of the effect of *C. difficile* exposure on colorectal cancer. For example, we identified sex as a potential effect modifier, which would not have been accounted for due to the matching system utilized in that study.

Our detected relationship between persistent *C. difficile* exposure and colorectal cancer diagnosis is important for several reasons. First, these findings confirm similar results observed in the mouse model work previously mentioned (13); in these models, colorectal tumorigenesis was observed only in mice with prolonged *C. difficile* toxin production, and it disappeared in mice treated with vancomycin to eradicate the pathogen. Additionally, there is growing awareness of difficulty with *C. difficile* eradication despite appropriate antibacterial therapy and improvement in clinical symptoms in humans. In fact, a majority of recurrent CDI (rCDI) episodes are suspected to be caused by reactivation of noneradicated bacterial spores as opposed to incident reinfection (14), and such chronic colonization and low-level toxin exposure could modify colorectal cancer risk. The incidence and consequences of failed eradication outside of a higher risk for rCDI are poorly studied in human hosts, but mouse models (13, 15) suggest that chronic enteric inflammation, dysbiosis, and possibly colorectal oncogenesis would be expected in susceptible hosts, potentially even in the absence of overt clinical symptoms of disease recurrence (i.e., subclinical infection). It is also known in human subjects that toxin production by *C. difficile*, sometimes at high levels, can occur in asymptomatic individuals (16).

Taken together, the above studies in mice and humans lead us to hypothesize that persistent colonization with toxigenic *C. difficile* leads to chronic, low-level toxin production and subclinical colon inflammation that contributes to colorectal cancer development in susceptible hosts. Persistence of *C. difficile* in human hosts following treatment attempts is a phenomenon suspected to be increasing in incidence since the early 2000s because of dynamic changes in the predominating circulating strain of the pathogen to one with relatively high sporulation and decreased maximal toxin production potential (17).

Additionally, our findings raise the possibility that host sex may influence the relationship between *C. difficile* exposure and colorectal cancer development. In our data, there seemed to be an association between *C. difficile* exposure of any magnitude and colorectal cancer development in females but not with males; however, this did not reach statistical significance. A growing body of literature implicates female sex as a risk factor for both primary (10) and recurrent (18) CDI. Mechanisms purporting to explain this disparity remain unsolved but are hypothesized to involve a higher degree of healthcare (and specifically antibiotic prescription) engagement amongst females (19) and/or an influence of circulating estrogen on the modulation of *C. difficile* activity and gut microbiome composition (20).

The association between nominal *C. difficile* exposure and proximal colon or rectal (but not distal colon) cancer is suggested by our data, although statistically significant differences were not observed. The relatively small numbers of precisely coded colorectal cancer location diagnoses in our datasets make it difficult to hypothesize further about the potential anatomical influence of *C. difficile*–induced carcinogenesis. Interestingly, in the groundbreaking publication by Drewes and colleagues (13), mice who suffered from *C. difficile*–induced colon carcinogenesis seemed to exclusively develop distal colon tumors.

Such findings gain an added layer of relevance in an era of rapidly expanding options for the prevention of rCDI. Since 2022, two novel, live stool-based products have been approved by the FDA for rCDI prevention as quality-controlled, standardized alternatives for fecal microbiota transplantation. These products have been shown to significantly reduce rCDI rates in high-risk populations compared with those who receive antibiotics alone (21), although rates of *C. difficile* “eradication” following these therapies have not yet been extensively investigated and the products remain expensive (∼$9,000 to $17,000/single administration). By advancing our understanding of who is at significant risk of long-term health complications (e.g., potentially colorectal cancer) from persistent *C. difficile* toxin exposure through population-level studies such as this, we strive to personalize the decisions to deploy more aggressive interventions to eradicate *C. difficile* while balancing the cost of these interventions. Importantly, there is strong precedent for the identification, treatment, and confirmation of eradication of another chronic enteric pathogen, *Helicobacter pylori*, for the prevention of gastrointestinal cancers (22). Although we acknowledge substantial differences in pathophysiology and the scientific evidence base between *H. pylori–* and *C. difficile–*induced oncogenesis, our findings raise the possibility that a similar conceptual framework may be applied to address the burden of colorectal cancer.

An important limitation of our study is the heterogeneity of follow-up time between the two databases integrated. In particular, the JHM database contains data from 2016 to 2024, a span of 8 years (compared with 23 years in the MM database). Conclusions regarding effects of certain exposures on the natural history of colorectal cancer, which can take up to 10 years to develop (23), may be dampened. However, it is notable that median follow-up time is comparable between the two sites and that similar patterns of exposure effects on colorectal cancer are noted. Although the median follow-up in our study was relatively short (approximately 4–5 years) and shorter than the typical latency period for colorectal cancer development, we still identified a statistically significant association between CDI and colorectal cancer after applying a 1-year lag. Sensitivity analyses using 3- and 5-year lags produced similar results, although with wider CIs. These findings suggest that the relationship may not be entirely dependent on long latency and could reflect effects on both early and later stages of the colorectal carcinogenesis pathway, including adenoma formation and progression. Inclusion in the JHM cohort required a positive microbiological test result at some point for entry, raising concern for underestimation of the nonexposed cohort. Additionally, the retrospective and EHR-based design of our study invites limitations inherent in this type of research. For example, although we required at least one recorded encounter for each individual at least 1 year after their entry into the cohorts, it is difficult to control for the quality of the follow-up encounter (e.g., physician visit vs. imaging study) to ensure equitable risk for *C. difficile* retesting and colorectal cancer diagnosis. We did not evaluate the effect of smoking and other behavioral risk factors, including diet and physical activity, nor did we evaluate recent hospitalizations or surgeries. Furthermore, larger datasets are needed to further explore racial and anatomic subgroup differences, as our current data were insufficient for racial subgroup analysis (Supplementary Table S1).

Our study has several strengths compared with prior studies. Most importantly, our analysis method used *C. difficile* test results to construct cohorts as opposed to ICD-10 coding for CDI, the latter of which likely underestimates the type of *C. difficile* exposure that mouse models suggest is biologically relevant for colorectal tumorigenesis (i.e., subclinical carriage). Additionally, our ability to stratify the nominal dose effect (i.e., multiply positive vs. singly or never positive) of toxigenic *C. difficile* exposure by tracking persistently or recurrently positive test results without needing to rely on ICD coding for rCDI permits a high-resolution investigation into the effects of chronic exposure on colorectal cancer development, a novel technique for this question. Furthermore, the use of time-varying exposure models with cumulative dose updates over time enabled us to fully leverage the available data and improve estimate precision. To our knowledge, no prior studies have examined the CDI–colorectal cancer relationship using this type of survival analysis with time-varying exposure, which we consider a key strength of our study.

### Conclusion

In summary, we have shown through this multicenter, retrospective cohort study that persistent exposure to toxigenic *C. difficile* is associated with the later development of colorectal cancer in US adults, an effect that may be amplified by female sex. These findings advance mouse model evidence of the role of *C. difficile* in effecting colorectal tumorigenesis and lay the groundwork for an enhanced awareness of and urgency in detecting and addressing chronic, subclinical *C. difficile* toxin production in individuals at heightened risk of colorectal cancer.

## Supplementary Material

Supplemental Table 1 Association between nominal dose effect of CD infection (0, 1 and > 1) and colorectal incidence by race, Michigan Medicine (2000-2023), Johns Hopkins (2016-2024)

## Data Availability

The data generated in this study are available within the article and its supplementary data files.
