# Supplementary material for: Association between Clostridioides difficile Test Positivity and Colorectal Cancer Incidence in a Multisite Hospital-Based Retrospective Cohort Analysis
Source: Cancer Res Commun. 2026 Apr 14;6(4):821–31. doi: 10.1158/2767-9764.CRC-25-0606 (PMC13077084; doi:10.1158/2767-9764.CRC-25-0606)
Supplement: Supplemental Table 1 — Association between nominal dose effect of CD infection (0, 1 and > 1) and colorectal incidence by race, Michigan Medicine (2000-2023), Johns Hopkins (2016-2024) [file crc-25-0606_supplemental_table_1_suppst1.docx]

| **Supplemental table 1. Association between nominal dose effect of CD infection (0, 1 and > 1) and colorectal incidence by race, Michigan Medicine (2000-2023), Johns Hopkins (2016-2024)** | | | | | | | | | |
| --- | --- | --- | --- | --- | --- | --- | --- | --- | --- |
| **Characteristic** | **Total (Combined)** | | | **Michigan Medicine (MM)** | | | **Johns Hopkins (JHH)** | | |
| **CAUCASIAN** | | | | | | | | | |
|  | **Events/py** | **HR (95%CI)** | **p-value** | **Events/py** | **HR (95%CI)** | **p-value** | **Events/py** | **HR (95%CI)** | **p-value** |
| CD = 0 | **169**/672839 | Ref | -- | **119**/631607 | Ref | -- | **50**/41232 | Ref | -- |
| CD =1 | **18**/114318 | 0.77 (0.47, 1.26) | 0.3 | **11**/104094 | 0.94 (0.51, 1.74) | 0.8 | **7**/10224 | 0.55 (0.25, 1.21) | 0.13 |
| CD>1 | **13**/69343 | 1.84 (1.04, 3.25) | 0.037 | **9**/66232 | 2.66 (1.34, 5.28) | 0.005 | **4**/3110 | 1.03 (0.37, 2.85) | >0.9 |
| Age |  | 1.04 (1.03, 1.05) | <0.001 |  | 1.05 (1.04, 1.06 | <0.001 |  | 1.01 (1.00, 1.03) | 0.089 |
| DM |  | 1.40 (1.04, 1.90) | 0.028 |  | 1.50 (1.06, 2.11) | 0.021 |  | 0.96 (0.47, 1.97) | >0.9 |
| IBD |  | 1.43 (1.01, 2.02) | 0.046 |  | 1.24 (0.85, 1.81) | 0.3 |  | 3.60 (1.66, 7.77) | 0.001 |
| FHCRC |  | 2.93 (1.99, 4.32) | <0.001 |  | 2.68 (1.75, 4.09) | <0.001 |  | 4.12 (1.64, 10.4) | 0.003 |
| Male |  | 1.29 (0.97, 1.70) | 0.076 |  | 1.40 (1.00, 1.96) | 0.05 |  | 1.02 (0.61, 1.70) | >0.9 |
| **BLACK** | | | | | | | | | |
| CD = 0 | **35**/10959 | Ref | -- | **15**/75979 | Ref | -- | **20**/19101 | Ref | -- |
| CD =1 | **6**/10959 | 0.17 (0.02, 1.21) | 0.076 | **3**/13053 | 0.55 (0.07, 4.17) | 0.6 | **0**/4868 | -- | -- |
| CD>1 | **1**/17921 | 3.50 (1.46, 8.37) | 0.005 | **1**/9662 | 5.14 (1.43, 18.5) | 0.012 | **3**/1297 | 1.83 (0.54, 6.23) | 0.3 |
| Age |  | 1.03 (1.01, 1.05) | 0.002 |  | 1.05 (1.02, 1.08) | <0.001 |  | 1.01 (0.98, 1.04) | 0.6 |
| DM |  | 1.72 (0.93, 3.18) | 0.084 |  | 1.26 (0.50, 3.17) | 0.6 |  | 2.44 (1.04, 5.72) | 0.04 |
| IBD |  | 1.15 (0.50, 2.65) | 0.7 |  | 1.23 (0.43, 3.50) | 0.7 |  | 2.61(0.59, 11.6) | 0.2 |
| FHCRC |  | 2.14 (0.76, 6.06) | 0.2 |  | 0.85 (0.11, 6.38) | 0.9 |  | 3.88 (1.12, 13.5) | 0.033 |
| Male |  | 0.93 (0.50, 1.74) | 0.8 |  | 1.43 (0.58, 3.56) | 0.4 |  | 0.66 (0.27, 1.60) | 0.4 |
| **OTHER** | | | | | | | | | |
| CD = 0 | **9**/51027 | Ref | -- | **5**/43860 | Ref | -- | **4**/7167 | — |  |
| CD =1 | **3**/8603 | 2.38 (0.62, 9.06) | 0.2 | **2**/7073 | 3.97 (0.76, 20.7) | 0.10 | **1**/1530 | 1.13 (0.13, 10.2) | >0.9 |
| CD>1 | 0/5082 | -- | -- | 0/4751 | -- | -- | 0/331 | -- | -- |
| Age |  | 1.03 (1.00, 1.06) | 0.075 |  | 1.08 (1.02, 1.13) | 0.005 |  | 4.01 (0.63, 25.6) | 0.14 |
| DM |  | 1.89 (0.56, 6.31) | 0.3 |  | 0.99 (0.21, 4.55) | >0.9 |  | 0.00 (0.00, Inf) | >0.9 |
| IBD |  | 0.62 (0.08, 5.05) | 0.7 |  | 0.81 (0.10, 6.85) | 0.8 |  | 0.00 (0.00, Inf) | >0.9 |
| FHCRC |  | 2.56 (0.31, 20.8) | 0.4 |  | 2.61 (0.31, 22.0) | 0.4 |  |  |  |
| Male |  | 1.30 (0.42, 4.07) | 0.7 |  | 1.15 (0.25, 5.23) | 0.9 |  | 1.56 (0.26, 9.40) | 0.6 |
| ^1^Multivariate cox proportional hazard adjusted for age, sex, DM, IBD, FHCRC; ^2^Hazard ratio and 95% Confidence interval modeled with Cox proportional hazard | | | | | | | | | |

**6.8 - Supplemental table 1. Association between nominal dose effect of CD infection (0, 1 and > 1) and colorectal incidence by race, Michigan Medicine (2000-2021), Johns Hopkins (2015-2022).** Total, MM, and JHM patient cohorts are stratified by chart-identified race (Caucasian, Black, or Other). Each of these race subcohorts is then stratified by nominal *C. difficile* exposure status as described in Table 3 and tested for association with incident CRC development.
